# Recommendations of the EANS Global and Humanitarian Neurosurgery Committee for sustainable neurosurgical partnerships in low-resource settings

**DOI:** 10.1016/j.bas.2025.105873

**Published:** 2025-11-14

**Authors:** Nicolò Marchesini, Vicki M. Butenschoen, Andreas K. Demetriades, Said Idrissa Ahmada, Kostantinos Gousias, Fazlul Hoque, Thomas Kapapa, Patrick D. Kamalo, Pablo González-López, Jesus Lafuente, Nigel Mendoza, Rupavathana Mahesperan, Wouter A. Moojen, Vincenzo Paternò, Ondra Petr, Wilco C. Peul, Lukas Rasulic, Nicephorus Boniface Rutabasibwa, Jamil Rzaev, Ellianne J. dos Santos Rubio, Abenezer Tirsit Aklilu, Jake Timothy, Massimiliano Visocchi, Oleksandr Voznyak, Enoch O. Uche, Magnus Tisell

**Affiliations:** a Global and Humanitarian Neurosurgery Committee, European Association of Neurosurgical Societies (EANS), Brussels, Belgium; b Department of Neurosurgery, Azienda Ospedaliera Universitaria Integrata di Verona, Verona, Italy; c Leiden University Medical Center, Leiden, the Netherlands; d Department of Neurosurgery, Technical University Munich, School of Medicine, Munich, Germany; e Department of Neurosurgery, Royal Infirmary Edinburgh, NHS Lothian, Edinburgh, United Kingdom; f Department of Neurosurgery (NED Institute), Mnazi MMoja Hospital, Zanzibar, Tanzania; g Department of Neurosurgery, Athens Medical Center, Athens, Greece; h Square Hospital, Dacca, Bangladesh; i Department of Neurosurgery, Ulm University Hospital, Ulm, Germany; j Blantyre Institute of Neurological Sciences, Department of Neurosurgery, Queen Elizabeth Central Hospital, Ministry of Health, Blantyre, Malawi; k Department of Neurosurgery, Hospital General Universitario Alicante, Alicante, Spain; l Department of Optics, Pharmacology and Anatomy, University of Alicante, Alicante, Spain; m Department Neurosurgery, Hospital Del Mar & Hospital QUIRON, Barcelona, Spain; n Department of Neurosurgery, Charing Cross Hospital Imperial NHS HCT, United Kingdom; o Department of Neurosurgery, Haukeland University, Bergen, Norway; p Department of Neurosurgery, University Neurosurgical Center Holland, Leiden University Medical Center, Haaglanden Medical Center, Haga Teaching Hospital the Hague, the Netherlands; q Department of Neurosurgery, International Neurosurgical Institute, Hannover, Germany; r Department of Neurosurgery, Medical University Innsbruck, Innsbruck, Austria; s Clinic for Neurosurgery, Faculty of Medicine, University Clinical Center of Serbia, Belgrade, Serbia; t Department of Neurosurgery, Muhimbili Orthopaedic Institute (MOI), Dar Es Salaam, Tanzania; u Department of Neurosurgery, Novosibirsk State Medical University, Novosibirsk, Russia; v Department of Neurosurgery, Curaçao Medical Center, Willemstad, Curaçao; w Division of Neurosurgery, Department of Surgery, Addis Ababa University, Addis Ababa, Ethiopia; x Department of Neurosurgery, Leeds General Infirmary, Leeds, United Kingdom; y Institute of Neurosurgery Fondazione Policlinico Gemelli, Largo Gemelli, 8, 00168, Rome, Italy; z Centre of Neurosurgery, Clinical Hospital “Feofaniya”, Kyiv, Ukraine; aa Division of Neurosurgery, College of Medicine, University of Nigeria Nsukka, Ituku, Enugu, Nigeria; ab Department of Neurosurgery, University of Gothenburg, Gothenburg, Sweden

**Keywords:** Global neurosurgery, Global health partnerships, Low- and middle-income countries, Sustainable collaborations, Consensus recommendations, European neurosurgery

## Abstract

**Background:**

Despite growing interest in global neurosurgery, equitable access to neurosurgical care in low- and middle income countries (LMICs) remains limited. European-led collaborations have received little attention, and practical guidance for initiating and maintaining partnerships is lacking.

**Research question:**

This study aimed to develop a set of consensus recommendations to guide sustainable neurosurgical partnerships between European institutions and partners in LMICs.

**Material and methods:**

Following a prospective qualitative study involving 14 neurosurgeons— seven from European institutions and seven from LMIC partners, key factors for successful institutional partnerships were identified. Evidence was synthesized into an initial list of 31 recommendations. Following consensus discussions at the 2024 EANS Congress and additional expert validation, 22 final recommendations were established.

**Results:**

Recommendations were organized into three phases: initiation, development, and maintenance. Key elements for initiation include building mutual trust, avoiding paternalistic dynamics, engaging local institutions, and defining shared goals. Development-focused recommendations highlight the importance of context-adapted, in-person training, bidirectional knowledge exchange, and inclusive program design. Long-term maintenance emphasizes self-sufficiency, equipment sustainability, continuous communication, and equity. A structured rating process ensured that each recommendation achieved at least 70% consensus among experts. Implementation insights were included as a supplement.

**Discussion and conclusions:**

This study presents the first structured, evidence-based recommendations tailored explicitly to European–LMIC neurosurgical collaborations. Emphasizing equity, sustainability, and local ownership, these guidelines offer a framework for future partnerships and may be relevant across other global health disciplines. Future validation in real-world settings will help refine their applicability and broaden their impact.

## Abbreviations:

**LMICs**: low- and middle-income countries
**HICs**: high-income country
**EANS**: European Association of Neurosurgical Societies

## 1 Introduction

Neurosurgical care remains highly inequitable in resource-limited settings, and the global neurosurgery subspecialty is gaining increasing attention as a means to address these disparities (Garcia et al., 2024). Many low- and middle-income countries (LMICs) lack essential infrastructure, medical equipment, and a sufficient number of trained neurosurgeons and supporting personnel/departments, leading to a significant burden of untreated neurosurgical patients (Haizel-Cobbina et al., 2024; Weiss et al., 2019). The challenges are further compounded by the migration of trained professionals, financial limitations due to poverty and funding problems, and the comparably high incidence of trauma and infectious diseases (Tropeano et al., 2019; GBD, 2016 Traumatic Brain Injury and Spinal Cord Injury Collaborators, 2019). Finally, cultural perceptions, low healthcare access awareness, and insufficient rehabilitation services further hinder effective neurosurgical care (Dewan et al., 2018; Meara et al., 2015a; Adegboyega et al., 2021).

Efforts to mitigate these issues have largely relied on bilateral partnerships between high-income countries (HICs) and LMICs, with a growing emphasis on fostering self-sustainable collaborations that empower LMIC institutions and reduce long-term dependency on external foreign support (Lepard et al., 2020; Fuller et al., 2021; Almeida et al., 2018; Timothy et al., 2022). The success of these initiatives is increasingly measured by their ability to establish independent and resilient neurosurgical training programs (Marchesini et al., 2024; Krakauer et al., 2024; Ukachukwu et al., 2022).

Despite growing interest in these partnerships, there has been limited exploration of European-led neurosurgical collaborations. Similarly, the critical pitfalls and success factors, derived from real-world experiences, are often overlooked (Marchesini et al., 2022). This lack of evidence results in insufficient guidance on the topic, potentially limiting the initiation of new projects or hindering the progress of existing ones.

Based on a recent qualitative analysis of the experiences of key collaborators from European neurosurgical institutions and their partners in LMICs, valuable insights on this issue were gathered (Marchesini et al., 2025). Drawing on this data, a set of recommendations has been developed as a possible guidance.

In this article we present the EANS (European Association of Neurosurgical Societies) Global and Humanitarian Neurosurgery Committee recommendations for initiating, developing, and maintaining collaborations between European institutions and partners in low-resource settings. In addition to general principles, we provide actionable insights to address common challenges and enhance the effectiveness of future global neurosurgical partnerships. The overall approach may be applicable to other medical specialties, offering useful guidance for similar initiatives.

## 2 Methods

**Fig. 1:**
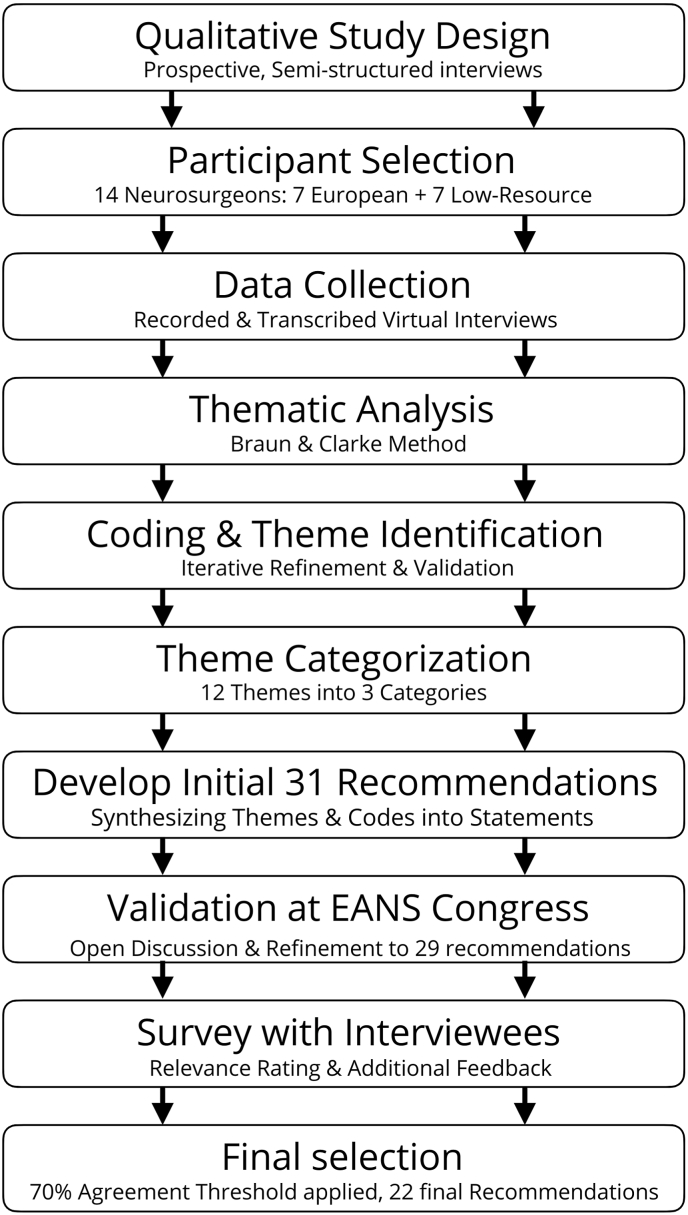
Synopsis of the process for developing the recommendations.

The methodology for developing the final recommendations included an initial qualitative analysis phase. A detailed description of the full qualitative methodology, compliant with the COREQ checklist, is reported in the primary qualitative paper; herein, we provide only the essential information to contextualize the subsequent consensus development process (Marchesini et al., 2025). After the qualitative study, a set of statements was developed and validated, based on the emerging data. A graphical summary of the process is shown in Fig. 1.

### 2.1 Qualitative analysis

The study employed a prospective qualitative research design to answer the following research question: What are the most salient interpersonal and systemic factors relevant for the a) initiation, b) development, and c) maintenance of effective and sustainable collaborations between European neurosurgical departments/organizations and Institutions/organizations in context with limited resources? As a first step, fourteen neurosurgeons were purposefully selected based on their direct involvement in such collaborations. To ensure diversity and balance, seven European partners were paired with their counterparts from neurosurgical institutions in resource-limited settings. Data collection involved semi-structured virtual interviews that were recorded and subsequently transcribed verbatim. For data analysis, thematic analysis was conducted following the framework proposed by Braun and Clarke (2006). The principal investigators reviewed the transcripts multiple times to achieve immersion and develop an understanding of the data. The process of open coding was used to generate a coding framework, which was refined iteratively through several cycles of coding and adjustments. Once the codes were established, they were grouped into themes based on patterns and relationships within the data. After the themes and codes were finalized, they were shared with the interviewees, who were invited to provide additional insights or clarifications, ensuring that the interpretations resonated with their experiences.

After qualitative data analysis, twelve themes were identified and, aligned with the research questions, they were categorized into three overarching categories: initiation, development and maintenance (Marchesini et al., 2025). Codes and themes represented a distillate of the experience of the interviewees and constituted the foundations for the subsequent phases of the process.

### 2.2 Development of the recommendations

Following the identification and finalization of the 12 core themes, the next step involved synthesizing these themes and associated codes into actionable statements or recommendations. This phase aimed to distill the complexities of the data into clear, practical guidance. The synthesis process began by grouping related codes under each identified theme, ensuring that patterns within the data were well captured. Each code was carefully reviewed to ensure its relevance and to identify any overlaps or gaps that could inform the final recommendations. The principal investigators then examined these groupings in depth, considering both the contextual factors within the interviews and the broader implications for collaboration. This allowed for the extraction of key insights from the data, which were framed as specific recommendations. Following this process, themes and codes evolved into an initial set of 31 distinct recommendations.

The methodology and results of the qualitative study were presented during a dedicated open session at the EANS Congress in Sofia (Bulgaria) on October 13th, 2024. The session provided an opportunity for all Congress participants to openly engage with the findings. Additionally, a total of eight members from the EANS Global and Humanitarian Neurosurgery Committee attended the session, contributing to the discussion and validation of the findings. During the session, the themes, codes, and derived 31 initial recommendations were presented in sequence, followed by extensive and open discussion among the attendees. Each recommendation was reviewed and refined, with the group openly working to achieve 100 % agreement on the formulation of each statement. This collaborative process led to the final refinement of 29 recommendations, down from an initial 31 statements.

After the session, between November and December 2024, the 29 refined statements were circulated to the 14 interviewed participants, who were asked to rate the relevance of each recommendation on a Likert scale (extremely relevant, very relevant, moderately relevant, slightly relevant, and not relevant). Additionally, participants were encouraged to provide further practical insights on how to implement the recommendations, allowing for an added layer of feedback and validation.

To ensure the final recommendations reflected consensus, a 70 % agreement about the degree of relevance rate for each recommendation threshold was applied, considering only “extremely relevant” or “very relevant” responses. This is a commonly used and pragmatically chosen cutoff in consensus studies and consensus-building exercises within medical education and global health, balancing the need for strong agreement with the recognition of diverse perspectives (Jünger et al., 2017; Noormahomed et al., 2024; Diamond et al., 2014). Statements below the threshold were omitted.

The 22 final recommendations were then grouped into three overarching categories—initiation, development, and maintenance, which provide structure to the final output. For completeness and transparency, omitted recommendations are also reported in the following sections and tables and clearly highlighted.

## 3 Results

**Fig. 2:**
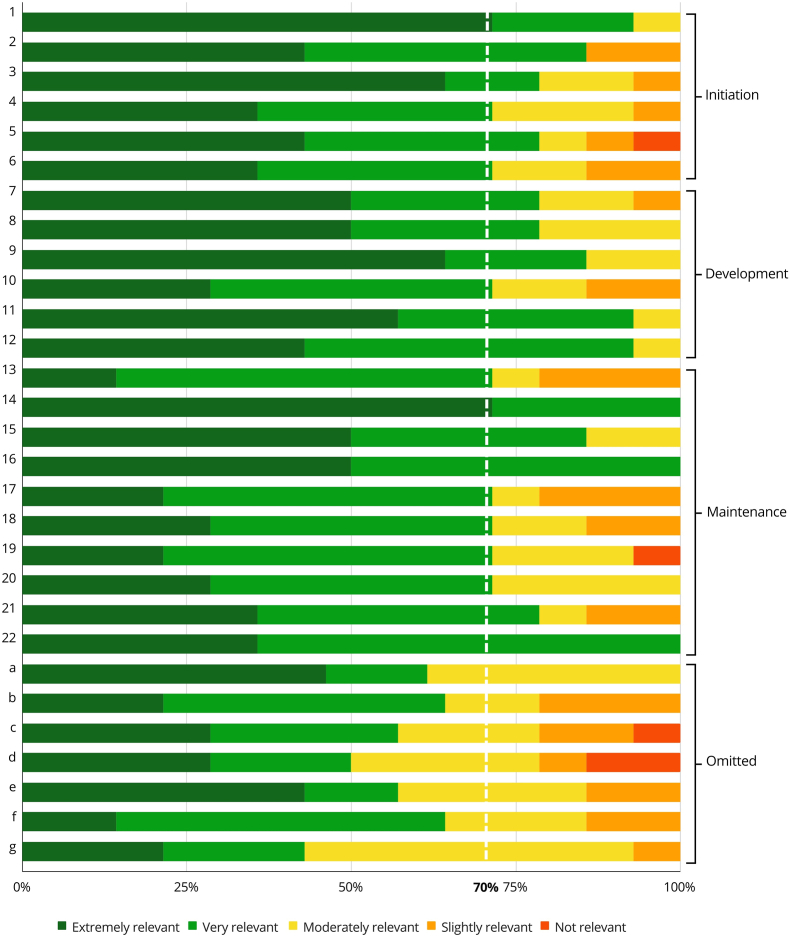
Graphical representation of the relevance ratings for the recommendations. Letters a to g indicate recommendations that did not reach the 70 % agreement threshold and were omitted. The dotted white line represents the 70 % threshold.

The results of the qualitative analysis phase have been published elsewhere (Marchesini et al., 2025). This section presents the panelists' quantitative ratings of the statements. The final recommendations for which consensus was reached are reported in Table 1, and separated into the three phases of collaboration: initiation, development, and maintenance. Table 2 includes the statements for which agreement was not reached. Fig. 2 graphically represents the relevance rating. Below, we present these recommendations contextualized with key qualitative insights that emerged from the interview data, illustrating how the lived experiences of partners directly informed these guidelines. Implementation insights, as suggested by participants during the rating phase and organized by the principal investigators, are included in Supplement 1. These insights were not subject to voting.

**Table 1:** Final statements and proportion of participants’ agreement leading to consensus or no consensus. Agreement is defined as a rating of “extremely relevant” or “relevant” for the specific recommendation.

No	Statement	Agreement rate
*Initiation*		
1	Prioritize the development of trust with local institutions through transparent, respectful, and culturally sensitive communication	93 %
2	Avoid preconceived judgments and hierarchical dynamics that may undermine local partners' autonomy and influence in the collaboration	86 %
3	Establish initial on-site contact early to build a strong personal and professional rapport between all stakeholders, ensuring commitment from both partners	79 %
4	Develop a joint plan with clear objectives and timelines, ensuring mutual agreement and understanding from the outset	71 %
5	Engage institutions and governments to secure partnerships agreements	79 %
6	Involve all partners in decision-making to ensure full commitment	71 %
*Development*		
7	Implement comprehensive training programs that focus on capacity building at all levels of care	79 %
8	Prioritize on-site training for the development of professional skills. Remote technologies are useful complements	79 %
9	Promote bi-directional professional exchanges	86 %
10	Prioritize academic collaborations and early exposure to research	71 %
11	Acknowledge and address infrastructure limitations and equipment challenges	93 %
12	Adapt training and clinical practice to local conditions and diverse cultural perspectives to healthcare	93 %
13	Ensure gender balance and diversity in neurosurgical training and professional development	71 %
*Maintenance*		
14	Focus on achieving progressive independence and long-term sustainability	100 %
15	Cultivate a sustainable system for supply and maintenance of equipment	86 %
16	Maintain clear and continuous communication between partners	100 %
17	Support international training and cross-specialty collaborations	71 %
18	Establish clear and measurable outcome indicators to track the program's success	71 %
19	Prioritize joint case selection to match available capacity and minimize risks	71 %
20	Develop strategies to recruit and retain staff	71 %
21	Address bureaucratic, legal, and medico-legal barriers, including issues related to travel, foreign neurosurgeon licensing, and liability protection for both local and international staff.	79 %
22	Address funding challenges by securing sustainable financial support	100 %

**Table 2:** Omitted recommendations as not reaching the 70 % agreement threshold.

No	Statement	Agreement rate
*Initiation*		
a	Adopt a tiered approach to collaborations, starting with achievable goals for future projects	50 % no agreement
b	Conduct assessments of the local context, including local needs, cultural dynamics, and critical stakeholders before formalizing the collaboration	64 % no agreement
c	Recognize and account for potential contextual challenges, including other competitive environments, language, the influence of colonialism and ethnic diversities	57 % no agreement
*Development*		
d	Design training curricula in collaboration with relevant local organizations	50 % no agreement
e	Ensure global visibility and recognition of achievements through publications and collaboration on research projects	57 % no agreement
f	Prioritize leadership training and decision-making support systems	64 % no agreement
*Maintenance*		
g	Encourage media involvement to raise awareness about the collaboration's progress and impact	43 % no agreement

### 3.1 Initiation

The themes of trust, equality, and joint planning dominated the initiation phase. Participants emphasized that trust is not an assumed principle, but should be built over time through personal connection and demonstrated respect.

The importance of trust directly informed Recommendation 1. As one partner from a low-resource setting stated, *“What can give you a strong foundation? Trust. And building trust takes time”*. This process requires what another neurosurgeon described as “*open, transparent, and culturally sensitive communication*".

To avoid undermining this trust, participants stressed the need for Recommendation 2, which advises avoiding paternalistic attitudes. A partner reflected this sentiment, stating, “*Bi-directionality means that there are certain rules … it's not a question of a unilateral relationship where decisions are taken from one center and the other just has to comply*”. Shared leadership is a prerequisite for sustainable collaboration.

Trust is most effectively built through early, in-person contact, as captured in Recommendation 3. One participant noted the importance of on-site visits to “*Go there and experience it yourself, really dive in*”. This allows collaborators to develop the professional and personal rapport essential for long-term commitment.

Once trust is established, a clear framework is critical and Recommendation 4 is a call for a jointly developed roadmap. This aligns with the experience of a partner who found that “*It is also important to have timelines. It doesn't have to be closed timelines. Things can be reevaluated and … modified*”. Furthermore, Recommendations 5 and 6 emphasize securing institutional support and involving all partners in decision-making to ensure full commitment and integrate the collaboration into broader health strategies.

### 3.2 Development

The development phase focuses on capacity building, adaptation, and mutual growth. The interviews revealed that effective development is not merely about transferring technical skills, but about building a resilient and context-aware system.

Recommendation 7 advocates for training that extends beyond surgical skills to all levels of care. This was powerfully summarized by a European neurosurgeon: “*You need to look at the other parts, like the nursing team, rehabilitation, and so forth … you can be the best surgeon in the world, and the patients will die as flies if you don't have good postoperative care*".

A dominant theme was the necessity to adapt to infrastructural and cultural contexts. Recommendation 11 directly addresses the critical challenge of infrastructure limitations, a reality captured in the simple but stark observation from a visiting surgeon: “*The electricity here is not stable. I did not know that when I came here. It broke down a lot of the things that I used*”. This necessitates Recommendation 12, which calls for tailoring training and practice to local conditions and cultural perspectives.

The principle of mutual benefit (Recommendation 9) was widely endorsed. Many interviewees acknowledged that European partners also gain from the exchange, operating and teaching in challenging circumstances. Integrating research (Recommendation 10) was seen as vital for sustainable academic growth, with one LMIC partner noting its importance for visibility and funding.

While remote tools are useful complements (Recommendation 8), on-site training was identified as most effective for hands-on skill acquisition. Finally, Recommendation 13 underscores the need for gender balance and diversity, ensuring equal opportunities for advancement in the profession.

### 3.3 Maintenance

The ultimate test of a collaboration is its long-term sustainability and transition to local independence. The interview data highlighted maintenance as a phase requiring continuous effort, strategic resource management, and clear metrics.

The overarching theme for maintenance is the transition to self-sufficiency, as outlined in Recommendation 14. This vision was echoed by an LMIC partner: “*You should have a timeline that a unit that is a recipient today … can become a donor tomorrow*”. This independence is underpinned by Recommendation 22, which stresses the need for diversified funding sources to ensure financial resilience beyond initial grants.

Long-term success depends on a reliable system for equipment and supply, as stated in Recommendation 15. A common pitfall was noted by a European partner: “*The second time I went over, the drills … were on the shelf gathering dust*”, emphasizing that donations must be paired with maintenance training. Furthermore, Recommendation 20 addresses the critical challenge of retaining trained staff to prevent brain drain, a frustration summed up by the observation that “*Human resources rapidly increase, but the available infrastructure does not … So that is frustrating … some of them leave for neighboring countries*".

Recommendation 16 highlights that maintenance requires clear and continuous communication to align visions and solve problems proactively, and cross-specialty interactions may be a valuable support, as highlighted in Recommendation 17. To track progress and ensure quality, Recommendation 18 calls for defining clear outcome measures. This is complemented by Recommendation 19, which advises careful joint case selection to match local capacity and minimize risks, especially in the initial stages.

Finally, Recommendation 21 acknowledges the bureaucratic, legal, and medico-legal barriers that can hinder collaborations, requiring clear legal frameworks and institutional agreements to facilitate smooth international partnerships.

## 4 Discussion

Our study offers a set of twenty-two practical recommendations for both LMIC and European partners to guide the initiation, development, and long-term sustainability of international neurosurgical collaborations. These recommendations were derived from qualitative data retrieved from balanced interviews with LMICs and European key partners actively engaged in successful global neurosurgical partnerships, ensuring that they reflect real-world experiences and challenges. To our knowledge, this study is the first systematic effort to establish recommendations specifically tailored to collaborations between LMICs and European global neurosurgery partners, and is well integrated into the ongoing discussions within the global neurosurgery agenda (Gupta et al., 2025). By categorizing the recommendations into three distinct phases—initiation, development, and maintenance—we offer a structured framework that emphasizes the importance of addressing the different stages of collaboration.

Our findings on the foundational role of trust and the imperative to avoid paternalism align strongly with the ongoing discourse on equitable partnerships in global health (Khan et al., 2021). The emphasis on mutual respect and culturally sensitive communication aligns with prior qualitative evidence identifying trust and transparency as cornerstones of global collaborations (Faure et al., 2021a). Furthermore, our consensus against power imbalances in collaborations directly engages with the urgent call to “decolonize global health” (Kumar et al., 2024). Our recommendations provide a practical framework for European institutions to operationalize these principles, by explicitly highlighting the importance of centering local autonomy and shared leadership from the outset.

Our first recommendation, focused on ensuring trust and culturally sensitive communication, has been highlighted in a study conducted in 2021 with interviews performed including scientists, researchers and research managers in international health collaborations (Faure et al., 2021b). This reinforces the idea that mutual respect and understanding should form the foundation of any collaboration, creating a solid foundation for future success. With over 90% of respondents rating it as very or extremely relevant, a consensual, transparent, and respectful partnership is essential for a successful and sustainable collaboration. This notion of sustainability extends beyond financial control and culminates in the final ownership of the collaboration by the LMICs counterpart, including the development of local expertise and infrastructure, formal transfer of project governance, and intellectual property. Ownership ensures interventions align with LMIC priorities and endure beyond external support.

To ensure long-term commitment, collaboration should extend beyond the leading hospital to include multiple institutions and seek governmental support. Broader engagement at multiple levels ensures that the program is integrated into the local healthcare system and increases its chances of sustainability. The benefits of the program for the local population should be acknowledged by political leaders, highlighting the need to invest in healthcare to achieve improved outcomes (Chikwari et al., 2024). Governments should not be the only actors in this process; the decision-making process should involve all partners engaged in the discussion. This inclusivity strengthens the shared ownership of the project and enhances accountability among all collaborators.

The importance of training is perhaps the most widely recognized recommendation for self-sustaining collaborations and its pivotal role has been extensively discussed in global health journals. The consensus on prioritizing on-site, comprehensive capacity building is indeed well-supported by existing literature on sustainable surgical workforce development (Mormina and Pinder, 2018; O'Flynn et al., 2022). While our panel affirmed that hands-on training in the local context is irreplaceable, this finding corroborates models of surgical education that stress the importance of training within the resource-constrained environment where skills will ultimately be applied, ensuring they are relevant and sustainable. The advocacy for bi-directional exchanges further enriches this model, acknowledging that HIC partners also gain invaluable experience and perspective, a benefit often understated in the literature but critical for fostering genuine mutual learning and respect (Timothy et al., 2022). Indeed, most interviewees recognized that European partners also benefit from going abroad, gaining more confidence and knowledge while operating and teaching in challenging circumstances. This two-way exchange enriches both parties, fostering a deeper understanding of different healthcare systems and enhancing professional development on both sides.

While our recommendations emphasize bilateral partnerships, scenarios involving multiple European institutions collaborating with a single LMIC partner necessitate additional coordination. Centralized communication channels—such as joint committees or shared digital platforms—should be established to align priorities, prevent duplication, and ensure the LMIC partner's voice remains primary in decision-making. This mitigates the risk of fragmented efforts or conflicting agendas.

Consistent with current literature, research partnerships must reject semi-colonial approaches—characterized by power imbalances where HIC investigators dominate priority-setting, methodology, and credit allocation, thereby undermining local autonomy and equitable recognition (Costello and Zumla, 2000; Paradie et al., 2022). To counter this, establishing transparent and culturally sensitive research practices is imperative, defined as: co-created agendas reflecting local health priorities, reciprocal capacity-building, contextual adaptation of protocols, and equitable authorship/benefit-sharing.

The overarching theme of transitioning toward local self-sufficiency is the cornerstone of sustainable program design and echoes the central tenet of the Lancet Commission on Global Surgery to strengthen health systems (Meara et al., 2015b). However, our study adds granularity by highlighting the often-overlooked systemic barriers to this goal. The critical challenges of equipment maintenance and staff retention identified by our participants are consistent with analyses of failed global health initiatives, where a lack of parallel investment in supply chains and professional incentives leads to the collapse of otherwise successful clinical programs (Marks et al., 2019; Dzwonczyk and Riha, 2012). For example, the introduction of navigation systems, robotics, and other high-maintenance equipment should be critically assessed and discussed with local partners beforehand. These discussions should focus on whether such technologies are appropriate given the local infrastructure, technical expertise, and financial resources. From the perspective of successful learning curves, training and the provided instruments should align with local conditions. Alternative protocols and adjustments to local circumstances are crucial, and constant feedback from both partners is necessary to ensure the ongoing relevance of the program. Iterative feedback loops are essential for refining strategies and ensuring that the program remains adaptable to evolving local needs.

Gender equality and diversity in LMICs remain unmet goals within the Global Surgery 2030 plan (Gender equity in global surgery). In our study, gender balance was rated as extremely or very relevant by approximately 70% of interviewees. This highlights the importance of addressing disparities in neurosurgery and ensuring equal opportunities for training and advancement. Issues related to the discrimination of women in the surgical field, despite the increasing number of women pursuing medical careers, should be addressed to ensure access to training and reduce barriers for potential female or diverse participants. This could be enhanced through focused and improved mentoring (Malik et al., 2021). Mentorship programs, tailored to address the unique challenges faced by marginalized groups, can play a crucial role in fostering inclusivity and diversity within the field.

While our recommendations reinforce universal principles of partnership, they carry distinct significance for European actors. The North American-led literature on global neurosurgery collaborations is more extensive, often involving different funding landscapes and institutional models (Almeida et al., 2018). European institutions, in contrast, often navigate more fragmented healthcare systems and diverse national policies. Our study, focused on partnership involving European Institutions, suggests that this diversity may be a strategic advantage. Experience with cross-border collaboration within the EU can be leveraged to manage the complex, multi-institutional partnerships often required in LMICs. Furthermore, the strong emphasis on sustainability and ethical collaboration in our findings aligns with core European values in development aid, offering a model that prioritizes long-term health system integration over short-term mission-based work.

The main strength of this study lies in its qualitative systematic design, which generated the foundations upon which the statements were built. The process enabled an in-depth exploration of interpersonal and systemic factors in a way that would not have been possible to assess with quantitative methods. By gathering insights from neurosurgeons with direct experience in such collaborations, the study provided a rich, real-world perspective on the challenges and strategies involved. The balance between European and low-resource participants is another strength that further validates the relevance of the recommendations. The iterative process of theme development, followed by validation through both EANS Congress discussions and feedback from interview participants, ensured that the final recommendations were widely relevant and grounded in expert consensus. The final recommendations reflect rigorous consensus criteria (Table 1). Though excluded from the final recommendations list (Table 2), items like media engagement frameworks, local context assessment (etc.) retain conceptual relevance. Additionally, the practical suggestions provided in the supplemental material can be useful for translating the developed statements into real-world implementation.

However, there are some limitations to consider. The sample size of 14 participants was determined by the qualitative methodology, aiming for rich, in-depth data from experts with specific experiences, and data saturation was achieved. For this initial effort, the focus was exclusively on neurosurgeons as they are typically the initiators and clinical leaders of such partnerships. This excluded the valuable perspectives of other critical team members, such as nurses, anesthesiologists, and hospital administrators, which should be a focus of future research to build a more holistic model. Moreover, the insights are based on interviews, which are subject to memory and biases. Furthermore, while personal experience is the essence of qualitative data, a potential limitation is the risk of social desirability bias, where expert participants may provide responses they perceive as aligning with ‘best practices' in global health. We excluded countries and regions affected by war, limiting our ability to draw conclusions about resource-constrained settings impacted by violence. Moreover, while the step at the EANS Congress provided valuable feedback, it may have been influenced by a specific group of specialists, potentially narrowing the diversity of viewpoints. Future developments should include a prospective analysis to evaluate the significance of the recommendations in real scenarios and a validation process for the statements beyond the neurosurgical field.

## 5 Conclusions

Through an iterative systematic process of synthesis, discussion, and validation, 22 recommendations for initiating, developing and maintaining collaborations between European and partners in low-resources settings were formulated. The statements provide practical guidance for enhancing future collaborations, emphasizing the importance of alignment, communication, and sustained commitment from all partners.

## Ethics approval and consent to participate

The study was conducted in accordance with the ethical principles of the Declaration of Helsinki. Participants’ consent was obtained by explicitly acknowledging voluntary participation and use of the data collected.

## Authors' contributions

Conception and design:

Acquisition of data: NM, VMB, all remaining authors.

Analysis and interpretation of data: NM, VMB, all remaining authors.

Manuscript draft: NM, VMB.

Critical revision for important intellectual content: all remaining authors.

Final approval: NM, VMB, all remaining authors.

## Declaration of generative AI and AI-assisted technologies in the writing process

During the preparation of this work, the authors used Chat GPT and Grammarly to revise the manuscript grammatically. After using this tool, the authors reviewed and edited the content as needed and they take full responsibility for the publication's content.

## Funding

This research received no specific grant from any funding agency in the public, commercial, or not-for-profit sectors.

## Competing interests

The authors declare that they have no competing interests.
